# Treatment of migraines with Tianshu capsule: a multi-center, double-blind, randomized, placebo-controlled clinical trial

**DOI:** 10.1186/s12906-019-2775-2

**Published:** 2019-12-16

**Authors:** Shengyuan Yu, Ye Ran, Wei Xiao, Wenjing Tang, Jianjun Zhao, Wei Chen, Huikui Zhuang, Cun Ouyang, Hai Lin, Dequan Liu, Tongjun Chen, Hui Huang, Baoshen Wang, Yanlei Hao, Zhongrui Yan, Shike Zhao, Yanling Wang, Jinjun Ni, Chaodong Wang, Wentao Ding, Guoqian Li, Jianhua Cao, Shujuan Tian

**Affiliations:** 1 Department of Neurology, Chinese PLA General Hospital, Fuxing Road 28, Haidian District, Beijing, 100853 China; 2 State Key Laboratory of New-tech for Chinese Medicine Pharmaceutical Process, Lianyungang, 222001 China; 3 Affiliated Hospital of Changchun University of Tradition Chinese Medicine, Changchun, Jilin China; 4 Brain Hospital of Jilin province, Changchun, China; 5 Second Affiliated Hospital of Shandong University of Tradition Chinese Medicine, Jinan, Shandong China; 6 Wuhan Sixth Hospital, Wuhan, Hubei China; 7 Xi’an Hospital of Tradition Chinese Medicine, Xi’an, Shaanxi China; 8 Langfang Tradition Chinese Medicine Hospital, Langfang, Hebei China; 9 Tangshan Tradition Chinese Medicine Hospital, Tangshan, Hebei China; 10 Huaibei People’s Hospital, Huaibei, Anhui China; 11 Luohe Hospital of Chinese Medicine, Luohe, Henan China; 12 Affiliation Hospital of Jining Medical University, Jining, Shandong China; 13 Jining No.1 People’s Hospital, Jining, Shandong China; 14 Ji’nan Municipal Hospital of Traditional Chinese Medicine, Ji’nan, Shandong China; 15 Cangzhou People’s Hospital, Cangzhou, Hebei China; 16 Kaifeng Hospital of Tradition Chinese Medicine, Kaifeng, Henan China; 17 Sanming First Hospital, SanMing, Fujian China; 18 Guang’anmen Hospital, China Academy of Chinese Medical Sciences, Beijing, China; 19 Quanzhou First Hospital, Quanzhou, Fujian China; 20 Yiyang Central Hospital, Yiyang, Hunan China; 21 The 260th Hospital of PLA, Shijiazhuang, Hebei China

**Keywords:** Tianshu capsule, Migraine, Herbal medicine, Multicenter, Randomized controlled trial

## Abstract

**Background:**

Tianshu capsule (TSC), a formula of traditional Chinese medicine, has been widely used in clinical practice for prophylactic treatment of headaches in China. However, former clinical trials of TSC were small, and lack of a standard set of diagnostic criteria to enroll patients. The study was conducted to re-evaluate the efficacy and safety of TSC post-marketing in an extending number of migraineurs who have diagnosed migraine with the International Classification of Headache Disorders, 3rd edition (beta version, ICHD-3β).

**Methods:**

The study was a double-blind, randomized, placebo-controlled clinical trial that conducted at 20 clinical centers in China. At enrollment, patients between 18 and 65 years of age diagnosed with migraine were assigned to receive either TSC (4.08 g, three times daily) or a matched placebo according to a randomization protocol. The primary endpoint was a relative reduction of 50% or more in the frequency of headache attacks. The secondary outcomes included a reduction in the incidence of headache, the visual analogue scale of headache attacks, days of acute analgesic usage, and percentage of patients with a decrease of 50% or more in headache severity. Accompanying symptoms were also assessed.

**Results:**

One thousand migraine patients were initially enrolled in the study, and 919 of them completed the trial. Following the 12-week treatment, significant improvement was observed in the TSC group concerning both primary and secondary outcomes. After therapy discontinuation, the gap between the TSC group and the placebo group in efficacy outcomes continued to increase. There were no severe adverse effects.

**Conclusions:**

TSC is an effective, well-tolerated medicine for prophylactic treatment of migraine, and still have prophylactic effect after medicine discontinuation.

**Trial registration:**

ClinicalTrials.gov Identifier: NCT02035111; Data of registration: 2014-01-10.

## Introduction

Migraine is defined as a recurrent common headache disorder. Commonly, the prevalence of migraine is estimated to be up to 9.3% per year, and the total annual financial cost of migraine is estimated at 47.8 billion USD in China [1]. Besides, migraine accounts for 5.6% of all years lived with disability (YLDs) in the world, and come second in the disability ranking (behind only low back pain) in the Global Burden of Disease (GBD) study of 2016. And what’s worse, migraine is the top cause of YLDs in the age group of 15–49 years [2, 3].

Considering the high headache frequency of patients with migraine, acute drugs are not often sufficient to control attacks. All migraine patients with frequent attacks should therefore consider pharmacological prophylaxis. Despite the substantial unmet needs, specific prophylactic treatment of migraine is still lacking [4]. Most of the prophylactic medications currently available were the result of the expansion of clinical indications for existing drugs. The following pharmacological classes are recognized as effective drug therapies for prophylactic treatment: antidepressants, antiepileptics, antihistamines, β-adrenergic receptor blockers, calcium ion channel antagonists, and onabotulinum toxin A [5].

The use of Tianshu prescription has a very long history in China in prophylactic treatment of headache. The formula of Tianshu Prescription was first recorded in *“Xuan Ming Lun Fang”*, Jin Dynasty (AD1115) [6]. Tianshu Prescription contains both Chuanxiong Rhizoma (CR) and Gastrodiae Rhizoma (GR), which are the dried rhizome of *Ligusticum chuanxiong Hort* and *Gastrodiae Elata Bl*, respectively (Table 1). The formulation of the Tianshu Capsule is according to *Pharmacopoeia of the People’s Republic of China (2015)*. The production process is as follows. The two herbs are crushed and refluxed with 90% ethanol twice, respectively. Then, the extract of the two herbs is combined and filtered. The filtrate is recovered ethanol and concentrated to obtain a transparent paste. At the same time, the dregs of the two herbs are boiled twice with water, respectively. Then, the decoction is combined and filtered. The filtrate is concentrated and mixed well with dextrin. After drying, the dextrin mixture added the above transparent paste is granulated, dried and put into capsules. It takes Chuanxiong Rhizoma 0.784 g and Gastrodiae Rhizoma 0.196 g to make one Tianshu capsule (TSC). Kanion Pharmaceutical Company produced both the TSC and placebo used in the study in December 2012. The batch number is 131211.

**Table 1:** Scientific species names of all ingredients of the Tianshu Capsule

English name	Latin name	Family name	Genus name
Rhizoma Chuanxiong	Ligusticum chuanxiong Hort	Umbelliferae	Ligusticum
Rhizoma Gastrodiae	Gastrodia elata Bl	Orchidaceae	Gastrodia

Gastrodia elata, one of the most important ingredients in TSC, is listed as a vulnerable plant on the IUCN Red List. Therefore, all of the Gastrodia elata used in medications are raised on professional production base farms rather than caught in the wild. Application of cultured Gastrodia elata in medication production is permitted by the government.

In traditional Chinese medicine, Chuanxiong Rhizoma was applied in the treatment of rheumatic disease, traumatic diseases, menstrual disorders [7], and migraine [8], while Gastrodiae Rhizoma was used alone or combined with other Chinese herbs to treat dizziness, paralysis, headache, and convulsion [6]. The herbal formulae have been used with clinical effects in the treatment of migraine, although the exact mechanisms are still unclear. Some researchers found that CR may target PTGS2, ESR1, NOS2, HTR1B, and NOS3 to regulate the vascular and nervous system, and GR may target other molecular to control migraine accompanying symptoms [9]. Xiaoping Sun et al. reported that Tianshu prescription could effectively reduce headache and prevent depression in nitroglycerin (NTG)-induced migraine rat model by mediating monoamine oxidase [10]. Jiao Guan et al. found that there were significant differences in pharmacokinetic properties of ferulic acid and gastrodin between normal and migraine rats after oral administration of TSC [11]. TSC, as a medicine for headache, was approved to go to the market in China in 2015 (Z10950004). Although TSC has been prescribed frequently by Chinese primary care physicians, the medication still lacks an evidence-based post-marketing reevaluation. Several clinical trials have been designed to evaluate the efficacy of TSC on migraine [12]. However, the sample sizes were small, diagnostic criteria of migraine and parameters for end-point observation were inconsistent, which cause the treatment results were too weak to draw reliable conclusions. Therefore, a prospective multi-center, randomized, double-blind, controlled clinical trial of TSC was conducted for post-marketing evaluation of safety and efficacy. This clinical trial had registered in ClinicalTrials.gov (NCT02035111).

## Methods

### Study design and oversight

A total of 20 clinical centers in China participated in this study. The research group was led by the Chinese PLA General Hospital which took responsibility for the work about the trial. The design of the project strictly complied with the “Guidelines for Controlled Trials of Drugs in Migraine: the third edition. A Guide for Investigators” [13]. The Ethical Committee of the Chinese PLA General Hospital approved this study (Authorized Document number C2013–066-01). Protocols in this study abide by the World Medical Association Declaration of Helsinki and China’s regulations and guidelines for good clinical practice. All subjects signed written informed consent before participating in this study. Kanion Pharmaceutical Company donated the study medication but had no other role in the study.

### Inclusion and exclusion criteria

All participants were between 18 and 65 years of age with a diagnosis of migraine with aura or without aura in line with the diagnostic criteria of the International Classification of Headache Disorders, third edition, beta version (ICHD-3β), and who had the ability to comprehend and to complete the research diary.

This study excluded patients who had the following characteristics: reported headache 15 days or more per month; suffered from a combination of other types of headaches, either simultaneously or at separate times; used prophylactic drugs during the last 3 months; used TSC in the last month; had resistance to acute analgesics; had severe comorbidities, including hypotension, severe infection, malignant tumors, cardio-cerebro-vascular diseases, or hepatic, renal, hematologic diseases; abused alcohol or other drugs; and pregnant women.

### Interventions

The study consisted of three phases: screening period lasting 4 weeks, treatment period lasting 12 weeks, and a follow-up period lasting 4 weeks. During the first period, all enrolled patients were screened by medical history, physical and laboratory examinations. During the treatment period, enrolled patients were randomly assigned in a 3:1 ratio to either accept TSC three times daily (4.08 g/d) or placebo of identical appearance with the same amount and frequency. It takes starch 0.318 g, sunset yellow 0.003 g, Melanin 0.002 g, and Tianshu capsule pre-granulation intermediate 0.017 g to make one placebo capsule. The placebo contains 5% capsule pre-granulation medium, which is the active ingredients of TSC, to ensure consistency of the smell and taste between TSC capsule and placebo. And the concentration of active ingredients in placebo is too low to have a treatment effect.

Researchers stopped drug treatment for patients with severe adverse events occurring during the period of trial. However, these patients were followed for safety assessment. Following the treatment period, we tracked the patients for 4 additional weeks. A detailed flow diagram of the experiment course is shown in Additional file 1: Figure S1. An independent statistician produced the randomization list for allocation. The computer-generated random medication code numbers were labeled on the study medication kit. The clinical researchers of each center were blind to the content of the distributed kit.

### Outcome measures

All patients were required to complete a daily headache diary throughout the entire trial, in conformity to the NINDS Common Data Elements [14]. The primary endpoint was a relative reduction of 50% or more in time during which a patient had a headache in the last 4 weeks of the 20-week trial compared to the 4-week baseline period. The secondary outcomes were reduction in the frequency of headache attacks per 4 weeks, the headache severity calculated by a visual analogue scale (VAS), days of acute analgesic usage, percentage of patients with a reduction of 50% or more in the severity of headache, and frequency of accompanying symptoms, which consisted of nausea, vomiting, photophobia and phonophobia from the 4-week baseline period to the final 4-week period of the trial. The interval between two episodes should be at least 24 h. Besides, the criterion for distinguishing between an extended episode and two episodes is that recurrence within 48 h after termination by sleep should be considered as one episode instead of two.

Safety assessment included physical examination, laboratory tests (blood routine, urinalysis, blood biochemistry) and electrocardiography, and the reports of adverse events (AEs). An AE record contained the date of onset and resolution, severity, duration, frequency, and relationship to study drug, action taken, and outcome.

### Statistical analysis

The sample size was calculated based on comparing the frequency of headache attacks between the TSC group and the placebo group. According to the previous study, the frequency of headache attacks decreased 4.52 ± 3.98 times in the TSC group compared with a decrease of 0.95 ± 1.19 times in the placebo group. A total sample size of 20 subjects (15 for TSC group, 5 for placebo group) was required to detect the difference, given type I error of 5% and power of the test of 90%. In order to get data on a large sample size in favor of a future study, we expanded the sample size to a total of 1000 subjects (750 for TSC group, 250 for placebo group). Our study is a post-marketing evaluation of the safety and efficacy of TSC. In the previous reports, the effect of the TSC group was better than that of the control group. The random distribution ratio of the sample size difference between groups was 3:1, which was more in line with the patient’s interests from the perspective of ethics.

Analyses of the primary and secondary outcome measurements were performed on the full analysis set (FAS) and per protocol set (PPS). For this study, the FAS population included all randomized subjects who accepted at least one dose of trial therapy and completed baseline estimation along with at least one post-treatment evaluation for the primary outcome measures. The missing values were replaced by the last observation. The PPS population contained subjects who fulfilled the 12-week treatment and 4-week follow-up observation as planned with no apparent protocol violations. Safety analyses were carried out on a safety set (SS) that consisted of all patients who had accepted at least one dose of the TSC and completed at least one post-treatment safety assessment.

The homogeneity of baseline characteristics between the two groups was analyzed with ANOVA or Pearson’s χ^2^ test. Safety analyses were performed on all randomized subjects who received at least one dose of study medication and at least one post-treatment safety measurement. Pearson’s χ^2^ test was used to analyze the incidences of AEs.

All statistical analyses were performed with SAS software, version 9.3 (SAS Institute, Cary, NC, USA). For data not complying with normal distribution, the nonparametric signed rank test was performed. The statistic procedures of paired t-test were employed to analyze continuous variables, which were expressed as mean ± SD. *P* values ≤0.05 were considered statistically significant (two-tailed).

## Results

### Patients

From April 2014 to March 2015, a total of 1000 migraine patients went through the screening process and were randomly selected to receive either TSC (*N* = 750) or placebo treatment (*N* = 250) for 12 weeks. The detail of recruitment, participation, and distribution of patients was demonstrated in Additional file 1: Figure S1. All of the 1000 randomized patients were included in the safety analysis. The PPS set contained 919 participants (TSC group, *n* = 690; placebo group, *n* = 229). No significant differences were observed between the two groups in demographic parameters, baseline headache characteristics (Table 2), and efficacy measurements (Table 3).

**Table 2:** Characteristics of patients receiving TSC and placebo

Characteristics		TSC (*N* = 750)	Placebo (*N* = 250)	*P* values
Age, mean ± SD, y		47.59 ± 11.86	47.82 ± 12.84	0.5957
Height, mean ± SD, cm		165.81 ± 6.89	165.61 ± 7.35	0.4332
Weight, mean ± SD, Kg		63.89 ± 9.32	63.57 ± 9.75	0.5717
Sex (%)	Male	277 (36.9%)	85 (34.0%)	0.4018
	Female	473 (63.1%)	165 (66.0%)	
Ethnic (%)	Han	745 (99.3%)	248 (99.2%)	1.0000
	Others	5 (0.7%)	2 (0.8%)	
Marital status (%)	Yes	714 (95.2%)	238 (95.2%)	1.0000
	No	36 (4.8%)	12 (4.8%)	
Profession (%)	Physical	149 (19.9%)	52 (20.8%)	0.7505
	Non-physical	601 (80.1%)	198 (79.2%)	
Women of child-bearing age (%)	Yes	221 (46.7%)	81 (49.1%)	0.5999
	No	252 (53.3%)	84 (50.9%)	
Menstrual cycle, mean ± SD, d		28.60 ± 2.47	28.56 ± 2.23	0.5932
Menstrual period, mean ± SD, d		5.25 ± 1.37	5.70 ± 1.48	0.0114
Diagnosis, no. (%)	Migraine with aura	200 (26.7%)	58 (23.2%)	0.2742
	Migraine without aura	550 (73.3%)	192 (76.8%)	
Headache attacks during the 3 months before screening period, mean ± SD, no.		8.81 ± 2.22	8.76 ± 2.33	0.7412
Allergic history, no. (%)	Yes	22 (2.9%)	3 (1.2%)	0.1012
	No	728 (97.1%)	247 (98.8%)	
Past medical history, no. (%)	Yes	57 (7.6%)	22 (8.8%)	0.5468
	No	693 (92.4%)	228 (91.2%)	
Use of headache treatment drugs, no. (%)	Yes	310 (41.3%)	103 (41.2%)	0.9704
	No	440 (58.7%)	147 (58.8%)	
With other diseases, no. (%)	Yes	54 (7.2%)	25 (10.0%)	0.1652
	No	696 (92.8%)	225 (90.0%)	

*d* day, *no.* number, *SD* Standard deviation, *y* years

**Table 3:** Baseline characteristics of efficacy measurements of patients receiving TSC and placebo

Characteristics	TSC (N = 750)	Placebo (*N* = 250)	*P* values
Times of headache attacks, mean ± SD, no.	4.01 ± 1.14	3.93 ± 1.15	0.2896
Headache duration, mean ± SD, h	8.26 ± 7.44	8.66 ± 8.75	0.4915
VAS of headache, mean ± SD	5.10 ± 1.41	5.00 ± 1.35	0.2940
Days of acute analgesic use, mean ± SD, d	2.55 ± 1.81	2.49 ± 1.85	0.6744
Nausea, mean ± SD, no.	3.59 ± 1.53	3.58 ± 1.49	0.6703
Vomiting, mean ± SD, no.	1.78 ± 1.95	1.76 ± 1.92	0.8831
Photophobia, mean ± SD, no.	2.02 ± 1.82	2.09 ± 1.84	0.6059
Phonophobia, mean ± SD, no.	1.40 ± 1.71	1.28 ± 1.62	0.4308

*d* day, *no.* number, *SD* Standard deviation, *VAS* Visual analogue score

### Primary outcomes

**Fig. 1:**
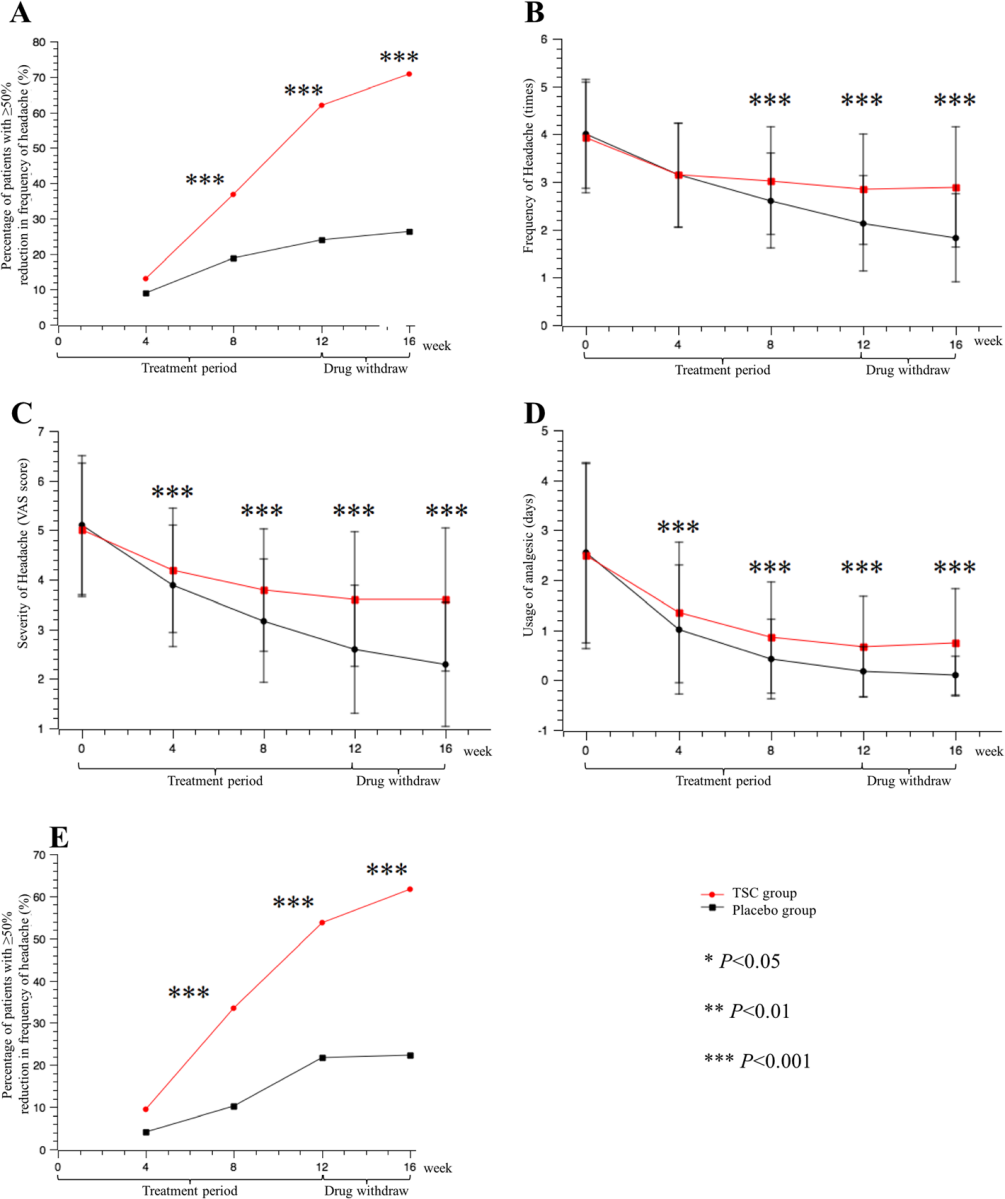
Primary and secondary outcome measures in the full analysis set (FAS). **a**: Percentage of patients with ≥50% reduction in frequency of headache; **b**: Frequency of headache; **c**: Severity of headache; **d**: Usage of acute analgesics; **e**: Percentage of patients with ≥50% reduction in severity of headache. The percentage of patients with ≥50% reduction in frequency of headache (**a**) increased gradually in the TSC group and was significantly different from the placebo group at week 8 (***P < 0.001). The frequency of headache (**b**) in the TSC group was significantly lower than in the placebo group after 8 weeks of treatment (***P < 0.001). The severity of headache (**c**) and usage of acute analgesics (**d**) were significantly lower in the TSC group than those in the placebo group after 4 weeks of treatment (***P < 0.001). The percentage of patients with ≥50% reduction in headache severity (e) was significantly increased in the TSC group compared with the placebo group after 8 weeks of treatment (***P < 0.001)

The percentage of patients who had a reduction of 50% or more in the frequency of headaches from the 4-week baseline period compared to the last 4 weeks of the 12-week treatment, was 62.1% in the TSC group compared with 23.9% in the placebo group (*P <* 0.0001; Fig. 1a; Table 4), in the FAS population. There was a significant difference in effect when the TSC was compared with the placebo. Also, after the 4-week follow-up observation after drug withdrawal, the gap between the two groups widened. The percentage of patients who had a reduction of 50% or more in the frequency of headaches increased to 70.8% in the TSC group compared with 26.3% in the placebo group at week 16 (*P <* 0.0001; Fig. 1a; Table 4).

**Table 4:** Change in efficacy measurements between TSC and placebo groups in the full analysis set (FAS)

Characteristics		TSC (*N* = 750)	Placebo (*N* = 250)	*P* values
Response rate (Percentage of patients with ≥50% reduction in frequency of headache), no. (%)	4 weeks	97 (13.0%)	22 (8.9%)	0.0794
	8 weeks	263 (36.7%)	46 (18.9%)	< 0.0001
	12 weeks	422 (62.1%)	56 (23.9%)	< 0.0001
	16 weeks	392 (70.8%)	60 (26.3%)	< 0.0001
Times of headache attacks per 4 weeks, mean ± SD	4 weeks	3.15 ± 1.09	3.15 ± 1.09	0.8647
	8 weeks	2.61 ± 1.00	3.02 ± 1.13	< 0.0001
	12 weeks	2.13 ± 1.00	2.85 ± 1.16	< 0.0001
	16 weeks	1.83 ± 0.92	2.89 ± 1.26	< 0.0001
VAS of headache, mean ± SD	4 weeks	3.88 ± 1.22	4.19 ± 1.25	0.0001
	8 weeks	3.17 ± 1.24	3.79 ± 1.23	< 0.0001
	12 weeks	2.59 ± 1.29	3.61 ± 1.36	< 0.0001
	16 weeks	2.29 ± 1.26	3.60 ± 1.45	< 0.0001
Days of acute analgesic use, mean ± SD, d	4 weeks	1.008 ± 1.29	1.350 ± 1.41	0.0003
	8 weeks	0.416 ± 0.80	0.856 ± 1.11	< 0.0001
	12 weeks	0.174 ± 0.51	0.671 ± 1.01	< 0.0001
	16 weeks	0.094 ± 0.38	0.754 ± 1.07	< 0.0001
Percentage of patients with ≥50% reduction in severity of headache, no. (%)	4 weeks	70 (9.4%)	10 (4.1%)	0.0044
	8 weeks	240 (33.5%)	25 (10.3%)	< 0.0001
	12 weeks	365 (53.7%)	51 (21.8%)	< 0.0001
	16 weeks	341 (61.6%)	51 (22.4%)	< 0.0001

*d* day, *no.* number, *SD* Standard deviation, *VAS* Visual analogue score

### Secondary outcomes

From the FAS analysis, the frequency of headache attacks per 4 weeks was significantly higher in the TSC group (2.61 ± 1.00) compared with the placebo group (3.02 ± 1.13) after the 8-week treatment course (*P* < 0.0001). With continued treatment, the frequency of headache in the TSC group was reduced to 2.13 ± 1.00, while the frequency in the placebo group remained relatively high at 2.85 ± 1.16 (*P* < 0.001) at the week 12. The severity of headache assessed by VAS scores was also significantly different between the TSC group (2.59 ± 1.29) and the placebo group (3.61 ± 1.36) at week 12 (*P <* 0.001; Fig. 1c; Table 4). The days of acute analgesic usage decreased to 0.17 ± 0.51 for the TSC group, while it was 0.67 ± 1.01 days for the placebo group at week 12 (*P <* 0.001; Fig. 1d; Table 4). Furthermore, the percentage of patients with a reduction of more than 50% in the severity of headache at week 12 was 53.7% in the TSC group and 21.8% in the placebo group (*P <* 0.001; Fig. 1g; Table 4). Additionally, during the experiment, the accompanying symptoms of migraine were also significantly diminished. During the last 4 weeks of the 12-week treatment, the frequency of nausea per 4 weeks decreased to 1.34 ± 1.19 in the TSC group versus 2.18 ± 1.49 in the placebo group (*P <* 0.001; Table 5); the frequency of vomiting decreased to 0.31 ± 0.78 in the TSC group versus 0.71 ± 1.29 in the placebo group (*P <* 0.001; Table 5); the frequency of photophobia decreased to 0.48 ± 0.99 in the TSC group versus 0.91 ± 1.39 in the placebo group (*P <* 0.001; Table 5); and the frequency of phonophobia decreased to 0.34 ± 0.87 in the TSC group versus 0.57 ± 1.19 in the placebo group (*P* = 0.002; Table 5).

**Table 5:** Change in accompanying symptoms between TSC and placebo groups in the full analysis set (FAS)

Accompanying symptoms		TSC (*N* = 750)	Placebo (*N* = 250)	*P* values
Nausea, mean ± SD, no.	4 weeks	2.50 ± 1.43	2.66 ± 1.47	0.1194
	8 weeks	1.89 ± 1.32	2.38 ± 1.47	< 0.0001
	12 weeks	1.34 ± 1.19	2.18 ± 1.49	< 0.0001
	16 weeks	1.06 ± 1.10	2.21 ± 1.59	< 0.0001
Vomiting, mean ± SD, no.	4 weeks	0.77 ± 1.22	0.93 ± 1.33	0.0523
	8 weeks	0.47 ± 0.97	0.83 ± 1.34	0.0002
	12 weeks	0.31 ± 0.78	0.71 ± 1.29	< 0.0001
	16 weeks	0.21 ± 0.70	0.79 ± 1.45	< 0.0001
Photophobia, mean ± SD, no.	4 weeks	0.98 ± 1.40	1.25 ± 1.58	0.0338
	8 weeks	0.69 ± 1.17	0.97 ± 1.39	0.0029
	12 weeks	0.48 ± 0.99	0.91 ± 1.39	< 0.0001
	16 weeks	0.42 ± 0.88	0.95 ± 1.51	< 0.0001
Phonophobia, mean ± SD, no.	4 weeks	0.67 ± 1.21	0.76 ± 1.31	0.3528
	8 weeks	0.44 ± 0.96	0.63 ± 1.23	0.0278
	12 weeks	0.34 ± 0.87	0.57 ± 1.19	0.0023
	16 weeks	0.33 ± 0.83	0.61 ± 1.19	0.0002

*no*. number, *SD* Standard deviation

After therapy discontinuation, the gap between the TSC group and the placebo group in secondary efficacy outcomes continued to increase. Four weeks after medication withdrawal, the frequency of headache attacks in the TSC group was reduced to 1.83 ± 0.92, far less than the frequency in the placebo group (2.85 ± 1.18, *P* < 0.001; Fig. 1a; Table 4). All of the secondary efficacy outcomes showed the same trend and were displayed in Fig. 1 and Table 4. Besides, the results of all of the primary and secondary outcomes in the PPS population coincided with that of the FAS population.

### Safety assessments

In the whole trial period, there were no clinically relevant alterations presented in the mean values of laboratory tests in all of the groups. None of the patients died during the experiment. There were 32 AEs that had been reported, among which 24 AEs occurred in the TSC group, and 8 AEs occurred in the placebo group. AEs that might be associated with treatment were observed in 9 subjects (1.2%) in the TSC group and 2 subjects (0.8%) in the placebo group (*P* > 0.05; Table 6).

**Table 6:** Patients experiencing AEs

Event	TSC (*N* = 750)	Placebo (*N* = 250)	*P* values
Adverse events, number of patients with event, no. (%)	24 (3.20%)	8 (3.20%)	1.0000
Possibly drug-related adverse events, no. (%)	9 (1.20%)	2 (0.80%)	0.7404
gastrectasia	1 (0.13%)	0	
stomach ache	1 (0.13%)	0	
abdominal tympany	2 (0.27%)	1 (0.40%)	
dizziness	2 (0.27%)	0	
conjunctival congestion	1 (0.13%)	0	
epistaxis	1 (0.13%)	1 (0.40%)	
menometrorrhagia	1 (0.13%)	0	
Adverse events resulting in experiment discontinuation, no. (%)	1 (0.10%)	1 (0.40%)	0.4377
epigastric pain	1 (0.10%)	0	
allergy with stomach ache	0	1 (0.40%)	
Serious adverse events, no. (%)	0	0	−/−

## Discussion

This study was the first multi-center, randomized, double-blind, placebo-controlled trial focusing on evaluating the efficacy and safety of the traditional Chinese medicine TSC for the prophylactic treatment of migraine. The results may provide a promising prophylactic treatment option for migraine. Moreover, the research methods utilized in this paper has great reference potential for similar migraine drug trials, including patient selection, outcome measures, and study duration.

Before our study, several clinical trials (a total of 10 studies encompassing 937 migraine patients) were carried out with relatively small sample sizes [12]. However, few studies adopted multi-centered, blind design methods, and it was challenging to control selection bias, and the placebo did not have an identical appearance with the same amount. Those factors led to a low positive predictive value. From this perspective, this rigorously designed multicenter, double-blind RCT was quite useful for providing accurate data for assessment of the effectiveness and safety of TSC for the prophylactic treatment of migraine.

The current study showed that TSC was superior to placebo for both the primary and secondary measurements, i.e., the response rate, frequency of headache attacks, headache severity (VAS scores), and the percentage of patients with ≥50% reduction in the severity of headache. Also, TSC considerably alleviated the accompanying symptoms in patients in the trial. Consequently, the usage of acute analgesics was reduced significantly as well.

The patients in this study well tolerated TSC. The reports of AEs in the TSC group were scarce after a 12-week continuous treatment. In particular, no hepatic or renal function damage was observed, which were frequently found in certain Chinese herbs. The most common AEs were increased menstruation and gastric discomfort.

Another valuable result that had never been reported before is that the parameters for evaluating pharmaceutical efficacy continued to improve after the medicine withdraw. This result suggested that TSC might have continuous effects on headaches prophylaxis after drug withdrawal by altering migraineurs’ brain function directly or indirectly, which implied that TSC might be safer than other prophylactic drugs with its longer prophylactic effect. It also reminded us to extend the follow-up period to assess the full treatment benefit further. The potential mechanisms involving in deserve further investigation.

Furthermore, we found that the placebo also affected headache remission. This effect may be because that headache was related to psychological conditions. It is therefore indicated that the inclusion of a placebo group is necessary for headache clinical trials.

Several limitations should be noted in the interpretation of this study. First, the scheme of the study ruled out high-risk individuals and those people with coexisting severe diseases; the results, therefore, could only reflect the features of specific populations of patients with migraine in China. Second, some criterions of diagnosis and tests tended to be subjective, as the severity of headache. Under ideal conditions, we should set up a pivotal group and lab to ensure the standard consistency of a variety of assessment methods. However, in order to guarantee the objectivity and correctness of the results, we recruited patients from 20 clinical centers in China. It was unrealistic to send all of the patients to one hospital. Third, we could not identify the role of depression and anxiety in variations of headache symptoms in this study, although several patients exhibited psychological characteristics suggestive of those statuses.

## Conclusion

This study demonstrated that TSC was an effective, safe, and well-tolerated therapy for patients with migraine and might have follow-up prophylactic function after drug withdrawal.

## Supplementary information

**Additional file 1.** Flow chart of experiment course.

## Data Availability

The datasets used and/or analyzed during the current study are available from the corresponding author on reasonable request.

## Abbreviations

AE: Adverse event
CR: Chuanxiong Rhizoma
FAS: Full analysis set
GR: Gastrodiae Rhizoma
ICHD-3β: International Classification of Headache Disorders, 3rd edition, beta version
PPS: Per protocol set
SS: Safety set
TCM: Traditional Chinese Medicine
TSC: Tianshu Capsule
VAS: Visual analogue score
