# Supplementary material for: Treatment of migraines with Tianshu capsule: a multi-center, double-blind, randomized, placebo-controlled clinical trial
Source: BMC Complement Altern Med. 2019 Dec 16;19:370. doi: 10.1186/s12906-019-2775-2 (PMC6915862; doi:10.1186/s12906-019-2775-2)
Supplement: Supplementary file 1 — Additional file 1. Flow chart of experiment course. [file 12906_2019_2775_MOESM1_ESM.docx]

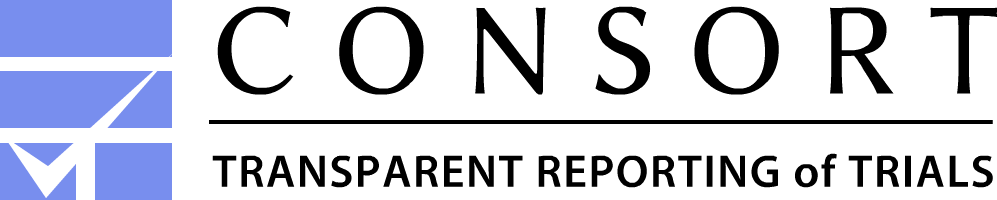


**CONSORT 2010 Flow Diagram**

Analysed (n= 690)
♦ Excluded from analysis (give reasons) (n=20)

Those patients did not observe the trial protocol.

Lost to follow-up (give reasons) (n= 40)

(We couldn’t contact the patients)

Discontinued intervention (give reasons) (n= 0)

## Analysis

Analysed (n= 229)
♦ Excluded from analysis (give reasons) (n= 8)

Those patients did not observe the trial protocol.

Lost to follow-up (give reasons) (n= 13)

(We couldn’t contact the patients)

Discontinued intervention (give reasons) (n= 0)

(One patient did not observe the trial protocol; the other declined to participate the trial)

Assessed for eligibility (n= 1000)

## Follow-Up

## Enrollment

Allocated to intervention (n= 750)

♦ Received allocated intervention (n= 750)

♦ Did not receive allocated intervention (give reasons) (n= 0)

## Allocation

Allocated to intervention (n= 250)

♦ Received allocated intervention (n= 250)

♦ Did not receive allocated intervention (give reasons) (n= 0)

Randomized (n= 1000)

Excluded (n= 0)

♦  Not meeting inclusion criteria (n= 0)

♦  Declined to participate (n= 0)

♦  Other reasons (n= 0)
